# Post-Progression Analysis of *EGFR*-Mutant NSCLC Following Osimertinib Therapy in Real-World Settings

**DOI:** 10.3390/cancers16142589

**Published:** 2024-07-19

**Authors:** Ilaria Attili, Carla Corvaja, Gianluca Spitaleri, Pamela Trillo Aliaga, Ester Del Signore, Antonio Passaro, Filippo de Marinis

**Affiliations:** Division of Thoracic Oncology, European Institute of Oncology, IRCCS, 20141 Milan, Italy; ilaria.attili@ieo.it (I.A.); carla.corvaja@ieo.it (C.C.); gianluca.spitaleri@ieo.it (G.S.); pamela.trilloaliaga@ieo.it (P.T.A.); ester.delsignore@ieo.it (E.D.S.); filippo.demarinis@ieo.it (F.d.M.)

**Keywords:** *EGFR*, chemotherapy, brain metastases, central nervous system, NSCLC, osimertinib

## Abstract

**Simple Summary:**

This study is a real-world study on a large cohort of patients with *EGFR*-mutant non-small-cell lung cancer (NSCLC) who progressed on osimertinib, focusing on access to subsequent treatments and progression patterns, including central nervous system (CNS) progression. Almost half of these patients had no access to further treatments due to worsening of clinical conditions or death. The outcomes of standard platinum-based chemotherapy were dismal and in line with those reported in the literature. Notably, intracranial disease progression was a rare event during osimertinib, whereas it occurred in half the patients during standard chemotherapy. Overall, the data highlight the clinical unmet needs in the treatment sequencing of single agent osimertinib and platinum-based chemotherapy alone, confirming the role of combination approaches and treatments in improving CNS control.

**Abstract:**

Background: Platinum-based chemotherapy is the current standard treatment option in patients with *EGFR*-mutant non-small-cell lung cancer (NSCLC) who progress on osimertinib. However, outcomes with chemotherapy are dismal, and the treatment of central nervous system (CNS) disease is an unmet need in this setting. Methods: Patients with *EGFR*-mutant NSCLC who were candidates to receive osimertinib in the metastatic setting at our Center from 2015 to 2022 were retrospectively evaluated to identify patients who received standard platinum-based chemotherapy post-osimertinib. Data were collected on treatment outcomes, with a focus on brain metastases and progression patterns. Results: A total of 220 patients received indication for osimertinib in the study period; *n* = 176 had adequate follow-up data. Overall, *n* = 117 patients experienced disease progression on osimertinib. The median time to osimertinib progressive disease (PD) was 15 months (95% confidence interval CI 13–18). Of them, 51 patients (45%) had no access to further treatments. Of the remaining patients, *n* = 8 received experimental treatments, and *n* = 55 received standard platinum-based chemotherapy and were considered for this study. Median duration of chemotherapy was 3 months (95% CI 2–5); the best responses among 53 evaluable patients were observed as follows: 15% partial response/complete response (PR/CR), 40% stable disease (SD), 45% PD. Median progression-free survival (PFS) and overall survival (OS) were 3 (95% CI 2–5) and 10 (95% CI 6–15) months, respectively. All patients had baseline and follow-up brain radiologic assessments, and n = 23 had brain metastases at the start of chemotherapy. With a median follow-up of 13 months, intracranial PD occurred in 47% patients, being the first site of PD in 59% of cases. The median time for intracranial (IC) PD was 2 months (95% CI 2–7). IC PD occurred as oligometastatic in 29%, whereas in 71% of cases, it was associated with systemic PD. Conclusions: Access to subsequent treatments and CNS progression are confirmed unmet needs in *EGFR*-mutant NSCLC patients. Clinical and CNS-specific outcomes in patients receiving standard chemotherapy after the failure of osimertinib are dismal. Novel upfront treatment options with demonstrated prolonged PFS and better CNS outcomes may help address this important issue.

## 1. Introduction

Genomic alterations in the epidermal growth factor receptor (*EGFR*) gene are found in approximately 15% of non-small-cell lung cancer (NSCLC) cases in the Caucasian and 50% in the Asian population [1,2,3]. Of them, 85–90% are represented by common actionable mutations (exon 19 deletions and exon 21 p.L858R point mutation) that are sensitive to treatment with EGFR tyrosine kinase inhibitors (TKIs) [4].

In patients with *EGFR*-mutated NSCLC, the current standard of care in the first-line setting is the third-generation EGFR tyrosine kinase inhibitor (TKI) osimertinib [5,6,7]. The same compound is also used in the setting of resistance to first- and second-generation EGFR TKIs, in the presence of the acquired p.T790M point mutation in the exon 20 of *EGFR* gene (around 60% of cases) [8,9].

Treatment with osimertinib allows high response rates, prolonged progression-free survival (PFS) and overall survival (OS) [5,7]. However, nearly all patients inevitably develop treatment resistance, due to the occurrence of different acquired resistance mechanisms and tumor heterogeneity [5,10,11,12,13,14].

The central nervous system (CNS) is commonly involved in *EGFR*-mutated NSCLC, with 25–40% of patients having brain metastases at diagnosis, and a further 20–30% of patients developing brain metastases during the disease course [15,16,17]. Third-generation EGFR TKIs have high CNS penetration, and osimertinib shows 66–70% intracranial response rate [18,19,20]. As such, the failure of osimertinib treatment may translate to failure of intracranial disease control.

Outside of clinical trials, platinum-based chemotherapy has represented for years the recommended next-line treatment after the failure of osimertinib, according to international guidelines [21]. The historical results of chemotherapy in this setting are dismal (median PFS 4.2–5.5 months) [8,22,23,24], leading to the search for novel combinations to overcome these limitations.

Very recently, the phase 3 MARIPOSA-2 trial results were presented, showing improved PFS with the combination of platinum-based chemotherapy and the bispecific antibody amivantamab in the osimertinib progression setting, compared to chemotherapy alone [23]. In this trial, the investigational combinations (amivantamab plus chemotherapy; amivantamab plus chemotherapy with lazertinib) showed a significant improvement of intracranial PFS, also confirmed in patients with untreated brain metastasis.

In parallel, platinum-based chemotherapy has been evaluated in combination with osimertinib in the front-line treatment in the phase 3 FLAURA2 trial, showing improved PFS over osimertinib alone, receiving approval by the Food and Drug Administration (FDA) for use in this setting [25].

A lively international debate is ongoing around the results of these trials, highlighting the potential limitations of applying combination regimens to all-comers *EGFR*-mutated NSCLC, in terms of increased toxicities, costs, and logistics, that may lead to restricting the adoption of these combinations.

The aim of our work is to evaluate the real-world outcomes of the osimertinib-progressing population in the current scenario, in order to highlight the clinical unmet needs in the treatment sequencing of single agent osimertinib and platinum-based chemotherapy alone.

## 2. Methods

We conducted a retrospective study on patients with *EGFR*-mutant NSCLC who were candidates to receive osimertinib in the metastatic setting at our Center from January 2015 to December 2022. Medical records of all patients with advanced *EGFR*-mutant NSCLC who were registered for osimertinib treatment in the study period were screened to identify patients with complete clinical, therapeutic, survival data, and adequate follow-up. In particular, the aim of our retrospective study was to describe the real-world outcomes, as well as the outcomes and progression patterns in the post-osimertinib setting. Hence, data were collected regarding disease progression on osimertinib, post-osimertinib-treatments, outcomes to standard chemotherapy with platinum plus pemetrexed, and brain metastases evaluation.

All the study procedures were carried out by the general authorization to process personal data for scientific research purposes from “The Italian Data Protection Authority” (http://www.garanteprivacy.it/web/guest/home/docweb/-/docwebdisplay/export/2485392, accessed on 10 January 2024). Anonymous numerical codes were used to manage all information regarding subjects, handled in compliance with the Declaration of Helsinki.

Median values were used to present continuous variables, and percentages (numbers) were used for categorical variables.

Real-world overall survival (rw-OS) and real-world progression-free survival (rw-PFS) were defined as the time between the start of osimertinib treatment and the occurrence of death from any cause and the time between the start of osimertinib treatment and progression or death from any cause, respectively.

In patients who received post-osimertinib chemotherapy treatment, PFS2 and OS2 were defined as the time between the start of chemotherapy and progression or death from any cause and the time between the start of chemotherapy and death from any cause.

Median survivals were estimated by using Kaplan–Meier methods. The reverse Kaplan–Meier method was used to calculate median follow-up. The Cox regression model was used for subgroup analysis on survival outcomes, and data were presented as hazard ratios (HRs) and their 95% confidence interval (CI), as appropriate. Continuous variables were compared through the Mann–Whitney test, whereas the two-sided chi-squared or Fisher exact tests were used to compare categorical variables, as appropriate.

Statistical significance level was set at *p* < 0.05 for all tests used. All statistical analyses were performed with R Studio (RStudio: Integrated Development for R. RStudio, Inc., Boston, MA, USA, v.4.1.2).

## 3. Results

### 3.1. Patient Population

**Figure 1:**
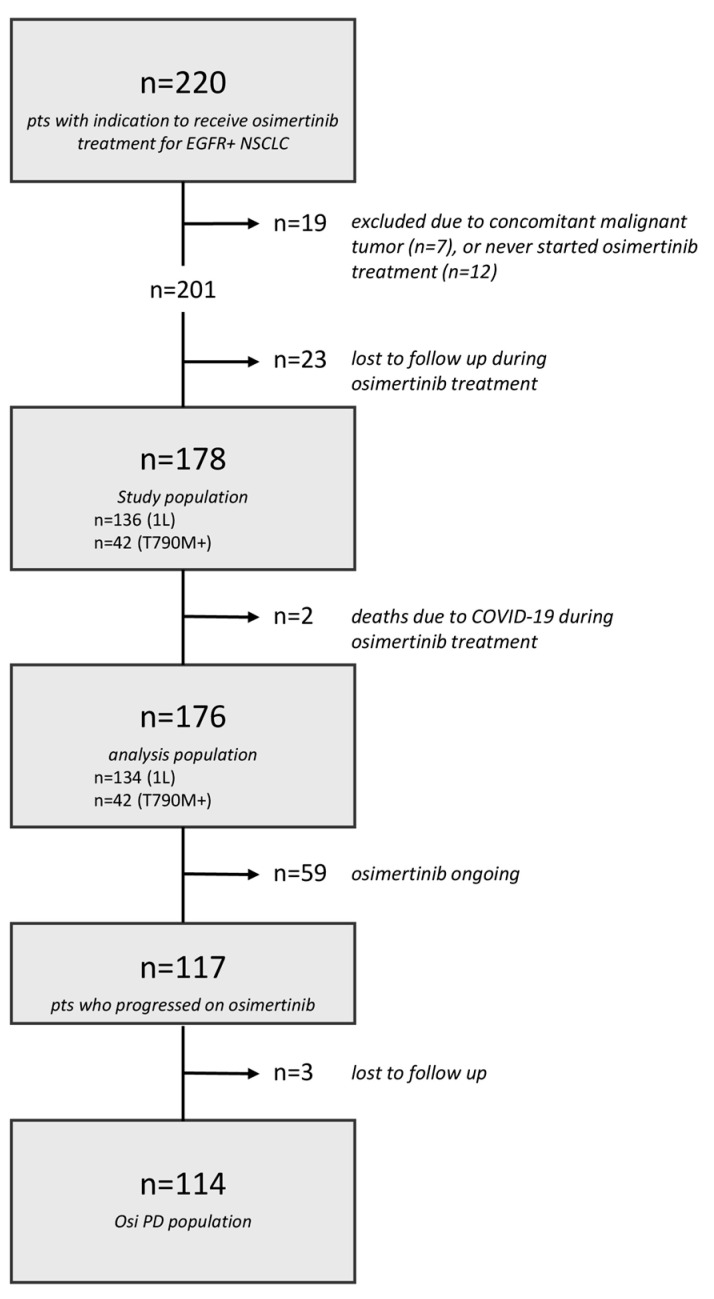
Flow-chart of study eligibility. A total of 220 patients receiving indication for treatment with osimertinib were screened. After applying selection criteria, 176 patients were considered as analysis population. Of them, after excluding 3 patients lost to follow-up after osimertinib progression, 114 patients were included in the osimertinib progressing population. Abbreviations: pts: patients; EGFR: epidermal growth factor receptor; NSCLC: non-small-cell lung cancer; 1L: first-line treatment; T790M+: presence of exon 20 p.T790M resistant mutation after failure of previous EGFR tyrosine kinase inhibitor; osi: osimertinib; PD: progressive disease.

Overall, 220 patients received indication for treatment with osimertinib in the study period at our Center. After a review of medical records, *n* = 7 patients were excluded because of concomitant second malignant tumor, and *n* = 12 never started osimertinib treatment. An additional 23 patients were lost to follow-up during osimertinib treatment, and *n* = 2 patients were excluded due to COVID-19-related death while on osimertinib, resulting in a total of *n* = 176 patients (analysis population) evaluated in this study (Figure 1).

#### 3.1.1. Real-World Outcomes in the Analysis Population

Among the *n* = 176 patients evaluated, median age at diagnosis of advanced/metastatic disease was 64 years (range 29–90), and 73% were females; most patients (65%) had Eastern Cooperative Oncology Group (ECOG) Performance Status (PS) 1 and were never-smokers (70.5%) (Table 1). Of them, *n* = 134 received osimertinib in the first-line setting (1L) and *n* = 42 in the T790M positive (T790M+) EGFR-TKI resistant setting.

Median follow-up from osimertinib start in the analysis population was 38 months (IQR 24–47). Overall, response rate with osimertinib was 70% (74% in the first-line setting, 57% in T790M+). Median duration of osimertinib treatment was 22 months (95% CI 18–28). In total, 29% of patients received local treatments for oligoprogressive disease on osimertinib with osimertinib continued. Of note, only 11 (6%) patients had CNS oligoprogression while on osimertinib (all received brain cyberknife radiation treatment).

Median progression-free survival with osimertinib (rw-PFS) was 22 months (95% CI 17–28) in the first-line setting and 26 months (95% CI 21–43) in the pretreated T790M+ setting.

Median overall survival with osimertinib (rw-OS) was 31 months (95% CI 26–43) in the first-line setting and 39 months (95% CI 22–NA) in the pretreated T790M+ setting (HR 0.84, 95% CI 0.52–1.35, *p* = 0.46) (Supplementary Figure S1).

#### 3.1.2. Osimertinib Progressive Disease (PD) Population

Overall, *n* = 117 patients experienced systemic disease progression on osimertinib. Of them, *n* = 3 patients were lost to follow-up after disease progression and were excluded from further analysis on the post-osimertinib setting (Figure 1).

Median age at osimertinib progression was 66 years (range 31–91). Median time to osimertinib progression was 15 months (95% CI 13–18) in the overall osimertinib PD population, 13.5 months (95% CI 11–17) in the first-line, and 21 months (95% CI 16–29) in the pretreated T790M+ setting.

At the time of disease progression leading to osimertinib discontinuation, 78.5% (*n* = 69) of 88 evaluable patients had systemic progression without CNS progression, 17% (*n* = 15) had systemic progression with CNS progression, while 4.5% (*n* = 4) had only CNS PD. No leptomeningeal metastases were observed.

Among patients with CNS disease at osimertinib start (*n* = 54, 31%), CNS progression occurred only in 25.5%.

**Figure 2:**
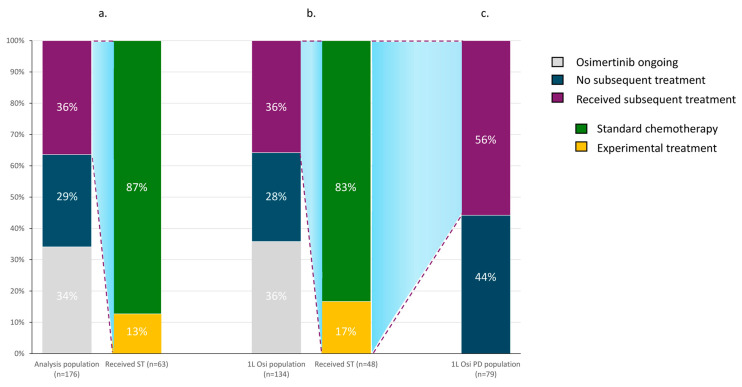
Subsequent treatments to osimertinib are shown. (**a**) Overall study population; (**b**) first-line osimertinib population; (**c**) first-line osimertinib-progressing population. Abbreviations: ST: subsequent treatments; 1L: first line; Osi: osimertinib.

Among the *n* = 114 patients in the osimertinib PD population (1L, *n* = 86; T790M+, *n* = 28), 44.7% (*n* = 51) did not receive any subsequent treatments (44% when limiting to the 1L setting) due to worsening clinical conditions and death. The remaining 55.3% patients (*n* = 63) received subsequent treatments: *n* = 55 received standard chemotherapy, and *n* = 8 received experimental treatments within clinical trials (Figure 2). Due to the risk for bias and the small numbers, the subset of patients receiving experimental treatments was not further evaluated.

### 3.2. Survival Outcomes with Standard Post-Osimertinib Chemotherapy

Among patients who received standard chemotherapy with platinum and pemetrexed after osimertinib failure (*n* = 55), median age was 61 years (range 44–83), and most patients (87%) had ECOG PS 1 (Table 1).

Median follow-up from the start of chemotherapy was 13 months (IQR CI 9–28), and median duration of chemotherapy was 3 months (95% CI 2–5). The best responses among 53 evaluable patients were observed as follows: 15% partial response/complete response (PR/CR), 40% stable disease (SD), and 45% PD.

With 85% (*n* = 45) of patients experiencing disease progression and 64% data maturity for overall survival, median PFS2 and OS2 were 3 (95% CI 2–5) and 10 (95% CI 6–15) months, respectively.

When looking at the previous osimertinib line, no statistically significant differences were observed in median PFS2 (1L: 4 months, 95% CI 2–6; T790M+: 2 months; 95% CI 0–7; HR 1.70, 95% CI 0.90–3.20, *p* = 0.12) and in median OS2 (1L: 10 months, 95% CI 7–NA; T790M+: 6 months, 95% CI 2–NA; HR 1.40, 95% CI 0.68–2.86, *p* = 0.37) (Supplementary Figure S2; Table 2). No statistically significant differences were observed according to the presence or absence of baseline brain metastases prior to the start of chemotherapy (Table 2).

### 3.3. CNS Progression Patterns with Standard Chemotherapy

All 55 patients had baseline and follow-up brain radiologic assessment (*n* = 35 computed tomography—CT scan, *n* = 20 magnetic resonance imaging—MRI). In total, 41.8% (*n* = 23) had baseline CNS metastases at the time of starting chemotherapy. Of them, *n* = 5 received brain locoregional treatment prior to starting osimertinib (cyberknife radiotherapy *n* = 4, whole brain radiation *n* = 1), *n* = 3 received previous brain radiotherapy for CNS oligoprogression on osimertinib, and *n* = 7 received brain radiotherapy prior to the start of chemotherapy.

**Figure 3:**
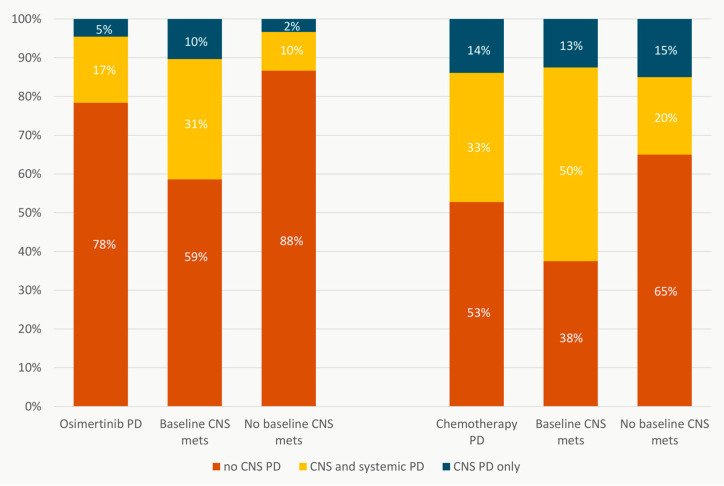
Progression patterns in patients progressing on osimertinib (left side) and in patients progressing on chemotherapy (right side). Abbreviations: CNS: central nervous system; PD: progressive disease; mets: metastases.

At the time of disease progression on chemotherapy (*n* = 45), 36 patients had evaluable radiological assessment: 33% (*n* = 12) had systemic progression with CNS progression, 14% (*n* = 5) had only CNS PD, while 53% (*n* = 19) had systemic progression without CNS progression (Figure 3). Among patients with CNS progression, leptomeningeal disease was radiologically suspected in three cases; however, clinical deterioration limited the possibility for cerebrospinal fluid (CSF) testing, feasible in only one case with cytological confirmation of leptomeningeal dissemination.

With a median follow-up of 13 months, intracranial PD (IC PD) occurred in 47% (*n* = 17) of the 36 patients with evaluable radiological assessment at disease progression, being the first site of PD in 59% of IC PD cases, symptomatic in 29%. Median time for IC PD was 2 months (95% CI 2–7). IC PD occurred as an oligometastatic event in 29%, whereas in 71% of cases, it was associated with systemic PD (Table 2).

The preferred approaches to treat IC PD were SBRT (12%), WBRT (12%), new systemic treatment (47%), and BSC (29%).

## 4. Discussion

Our study presents a real-world scenario of osimertinib use in clinical practice in patients with NSCLC with activating EGFR mutations, with a focus on post-progression patterns and outcomes.

Baseline demographic characteristics in our study population were comparable with that of pivotal clinical trials. However, about 10% of patients had an ECOG PS 2 at presentation. Overall, a similar number of patients had baseline brain metastases at osimertinib start (30.7%); for comparison, in the osimertinib arms of clinical trials, 19% in first-line FLAURA, 33% in the T790M+ AURA3 trial, 40% in the FLAURA2, and 41% in the MARIPOSA had baseline brain metastases [5,8,25,26,27].

In our real-world experience, with a comparable median follow-up of 38 months, responses and survival with osimertinib treatment were in line with those expected from the pivotal clinical trials [7,8,26], although even numerically longer rw-PFS and rw-OS were observed in the T790M+ cohort. This controversial observation might be due to the small size and longer observation period of the T790M+ population (osimertinib was temporally first approved in the pretreated setting); indeed, the difference observed has no statistical significance.

Of note, among patients who progressed on osimertinib, 44% did not have access to a further treatment line due to worsening clinical conditions and death. In the FLAURA trial, the data on post-progression treatment are reported considering the overall population, including those patients who were still on study treatment, showing 31% of patients in the osimertinib arm who did not receive a subsequent therapy [7]. However, when excluding patients still on study treatment (22%) [7], the patients who do not receive subsequent treatments are calculated into 39% of those who progress to osimertinib. Due to the absence of biomarker-guided treatment options, *EGFR* retesting was not routinely performed at osimertinib PD, so that no exploratory analyses are available based on the persistence or absence of *EGFR* mutation or different resistance mechanisms.

Moving to the population who received standard platinum plus pemetrexed in our study, we observe that median age was lower (61 years), and there were no patients with PS 2. Conversely, 41% of patients had brain metastases at chemotherapy start (all were treated prior to chemotherapy initiation). These data are comparable with that of the chemotherapy arm in both the AURA3 and MARIPOSA-2 trials [8,23].

The response rate observed with platinum plus pemetrexed chemotherapy in our cohort was 15%, numerically lower compared to about 30% in historical data [8,23,28], but limited by the small number of patients, and possibly in part related to personalized doses of chemotherapy administered compared to those administered in clinical trials.

In patients progressing after first-line osimertinib, median PFS with chemotherapy was 4 months, very similar to the results reported in the chemotherapy arm of the MARIPOSA-2 trial (median PFS 4.2 months) [23]. OS results in the MARIPOSA-2 trial are not mature and cannot be used as historical comparison for our data, whereas OS in the AURA3 trial is related to patients who could receive osimertinib after failure of chemotherapy [23,29].

The focus on CNS progression patterns highlighted the absence of efficacy in brain disease control with chemotherapy, with almost half the patients (47%) having CNS progression while on standard chemotherapy, whereas most patients (78.5%) who progressed on osimertinib did not show CNS PD (Figure 3). Due to the demonstrated CNS disease control upon osimertinib treatment, the possibility to add chemotherapy and continuing osimertinib at systemic disease progression, especially in the absence of CNS PD, is currently being explored [30]. Also, some data are emerging on the potential role of adding bevacizumab to osimertinib in patients with brain metastases [31]. However, such treatment options were not available in Italy outside of clinical trials; therefore, we could not explore the real-world results of such an approach for CNS outcomes.

In this view, when looking at recently presented data, the combination of amivantamab and platinum-based chemotherapy demonstrated significant reduction in the risk of CNS progression as compared to chemotherapy alone (HR 0.55, 95% CI 0.38–0.79) in the post-osimertinib setting in the MARIPOSA-2 trial [23]. In the front-line setting, the combination of chemotherapy and osimertinib also reduced the risk of CNS PD as compared to osimertinib alone (HR 0.58, 95% CI 0.33–1.01), with an observed intracranial response of 73% in the FLAURA2 study [32]. Of note, the improved PFS benefit of the combination in this trial appeared greater among patients with baseline brain metastases (median PFS 24.9 vs. 13.8 months; HR 0.47, 95% CI 0.33–0.66) [25].

As a further potential treatment option in the *EGFR*-mutated NSCLC setting, the role of antibody drug conjugates (ADCs) also deserves to be discussed, with patritumab-deruxtecan and datopotamab-deruxtecan showing response rates of about 30% to 43% in phase 2 trials conducted in heavily pretreated patients harboring actionable gene mutations [33,34,35]. In the phase 2 study HERTHENA-Lung 01 in the heavily pretreated setting (both EGFR TKI and platinum-based chemotherapy), the anti-HER3 ADC patritumab-deruxtecan showed encouraging intracranial activity, with a 33% CNS response rate in patients with non-irradiated brain metastases [33,36].

Of note, all the above-mentioned novel combination options are based on the agnostic approach, meaning that they do not require a tumor rebiopsy to give information on resistance mechanisms to osimertinib. Conversely, the biomarker-based approach is another line of investigation in the osimertinib-resistant setting, evaluating treatment combinations based on resistance mechanisms identified upon tumor rebiopsy [10,37]. Indeed, different resistant mechanisms and tumor heterogeneity could also be responsible for different treatment outcomes with agnostic approaches, including chemotherapy alone [38,39,40]. Due to the absence of standard biomarker-based treatment options at our center, our study population was not tested for resistance mechanisms in clinical practice; therefore, this information is not available for the study cohort.

Despite the limitations of a single-institution retrospective study, with risks of selection and information biases and findings that might not be generalizable to broader populations or different healthcare settings, we have reported on a large cohort of patients with NSCLC and *EGFR* mutations who received osimertinib treatment according to clinical practice. Multicenter data could provide a more comprehensive view on the research objects; however, the patients’ characteristics and real-world results for the overall population are in line with that of registrative clinical trials, giving strength to the validity of our results, especially with focus on the most represented first-line osimertinib population. The results observed in the post-progression setting are based on a relatively small but representative population, highlighting the unmet needs in treating *EGFR*-mutated disease. Of note, the subgroup analyses were performed in our study only as exploratory evaluations and not intended to inform any conclusion, as the small size of subgroups might limit the power to detect meaningful differences.

## 5. Conclusions

In conclusion, addressing the challenges of accessing subsequent treatments and effectively controlling CNS disease stand out as crucial unmet needs for patients undergoing osimertinib treatment with *EGFR*-mutated NSCLC. Furthermore, the outcomes related to both overall survival and CNS-specific responses in individuals transitioning to standard platinum-based chemotherapy post-disease-progression on osimertinib have been disheartening. Consequently, promoting the adoption of combination regimens, proven to extend overall PFS and effectively manage CNS disease, is imperative. These strategies should be considered for integration into the front-line treatment approach to maximize the postponement of initial PFS and mitigate the associated heightened risk of discontinuity in subsequent treatments.

## Figures and Tables

**Table 1:** Study population.

	Overall Population *n* = 176	Osimertinib PD Population *n* = 114	Standard Chemotherapy Population *n* = 55
Age			
median (range)	64 (29–90)	66 (31–91)	61 (44–83)
Sex (*n*, %)			
male	47 (26.7)	34 (29.8)	15 (27.3)
female	129 (73.3)	80 (70.2)	40 (72.7)
ECOG PS (*n*, %)			
0	43 (24.4)	27 (23.7)	7 (12.7)
1	114 (64.8)	74 (64.9)	48 (87.3)
2	19 (10.8)	13 (11.4)	0 (0)
Smoking status (*n*, %)			
never	124 (70.5)	74 (64.9)	40 (72.7)
former/light	37 (21.0)	26 (22.8)	13 (23.6)
current	15 (8.5)	14 (12.3)	2 (3.6)
*EGFR* mutation			
exon 19 deletions	109 (61.9)	67 (58.8)	35 (63.6)
exon 21 p.L858R	58 (33.0)	41 (35.9)	18 (32.7)
uncommon/compound	9 (5.1)	6 (5.2)	2 (3.6)
Osimertinib setting (*n*, %)			
1L	134 (76.1)	86 (75.4)	40 (72.7)
T790M+	42 (23.9)	28 (24.6)	15 (27.3)
Baseline CNS metastases (*n*, %)			
yes	54 (30.7)	37 (32.5)	23 (41.8)
no	122 (69.3)	77 (67.5)	32 (58.2)
Previous brain M treatment (*n*, %) ^§^			
no	33 (61.1)	25 (67.6)	10 (43.5)
yes, SBRT/Cknife	15 (27.8)	12 (32.4)	10 (43.5)
yes, WBRT	5 (9.3)	6 (2.7) °	3 (13.0)
yes, neurosurgery	1 (1.9)	0 (0)	0 (0)

Due to rounding, percentages may not always appear to add up to 100%. ^§^ percentage of those with baseline CNS metastases. ° Five patients who previously received WBRT received SBRT/Cyberknife, One patient who previously received Cyberknife received WBRT. Abbreviations: PD: progressive disease; PS: performance status; CNS: central nervous system; M: metastases; SBRT: stereotactic body radiation treatment; Cknife: cyberknife; WBRT: whole brain radiation treatment.

**Table 2:** Survival and CNS results in chemotherapy population.

	Patients *n* = 55	Patients with Baseline CNS Metastases *n* = 23	Statistical Testing
PFS2			HR 1.61 (95% CI 0.66–3.92)
months, median (95% CI)	3 (2–5)	3 (2–5)	
1L	4 (2–6)	4 (2–NA)	
T790M+	2 (0–7)	2 (2–NA)	
OS2			HR 1.05 (95% CI 0.53–2.06)
months, median (95% CI)	10 (6–15)	11 (5–NA)	
1L	10 (7–NA)	11 (7–NA)	
T790M+	6 (2–NA)	6 (3–NA)	
IC PD (*n*, %)	17 (31)	10 (43.5)	
brain only	5 (29)	2 (20)	*p* = 0.29
with systemic PD	12 (71)	8 (80)	*p* = 0.60
CNS metastases as first PD site			
yes (*n*, %)	10 (49)	4 (40)	
no (*n*, %)	7 (41)	6 (60)	*p* = 0.34
CNS metastases symptomatic			
yes (*n*, %)	5 (29)	2 (20)	
no (*n*, %)	12 (71)	8 (80)	*p* = 0.60

Abbreviations: CNS: central nervous system; PFS2: progression-free survival 2; OS2: overall survival 2; 1L: first-line osimertinib; T790M+: osimertinib administered in EGFR TKI T790M+ resistant setting; IC: intracranial; PD: progressive disease; HR: hazard ratio; *p*: *p*-value.

## Data Availability

The raw data supporting the conclusions of this article will be made available by the authors on request.

## Supplementary Materials

The following supporting information can be downloaded at: https://www.mdpi.com/article/10.3390/cancers16142589/s1, Figure S1: Survival outcomes with osimertinib in study population: Progression free survival (PFS) in the overall population (a) and according to treatment line (b); Overall survival (OS) in the overall population (c) and according to treatment line (d). Abbreviations: 1L: first line treatment; T790M+: presence of exon 20 p.T790M resistant mutation after failure of previous EGFR tyrosine kinase inhibitor; Figure S2. Survival outcomes in patients receiving standard chemotherapy (n = 55): a. Progression free survival (PFS); b. Overall survival (OS).
