# Supplementary figures and images for: Post-Progression Analysis of EGFR-Mutant NSCLC Following Osimertinib Therapy in Real-World Settings
Source: Cancers (Basel). 2024 Jul 19;16(14):2589. doi: 10.3390/cancers16142589 (PMC11274531; doi:10.3390/cancers16142589)

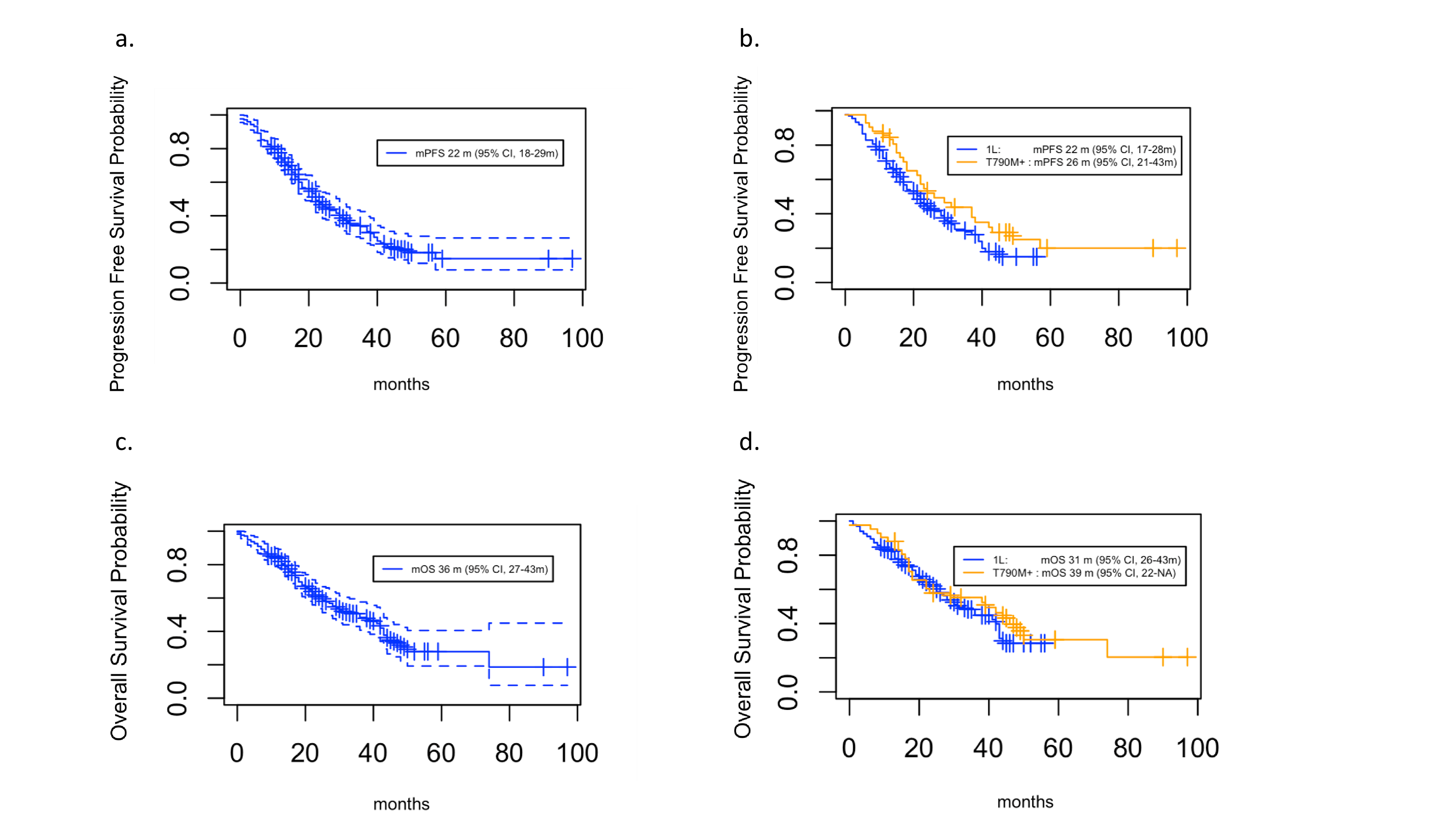

Supplement: Supplementary file 1 [file cancers-16-02589-s001.zip › Figure S1 egfr.tiff]

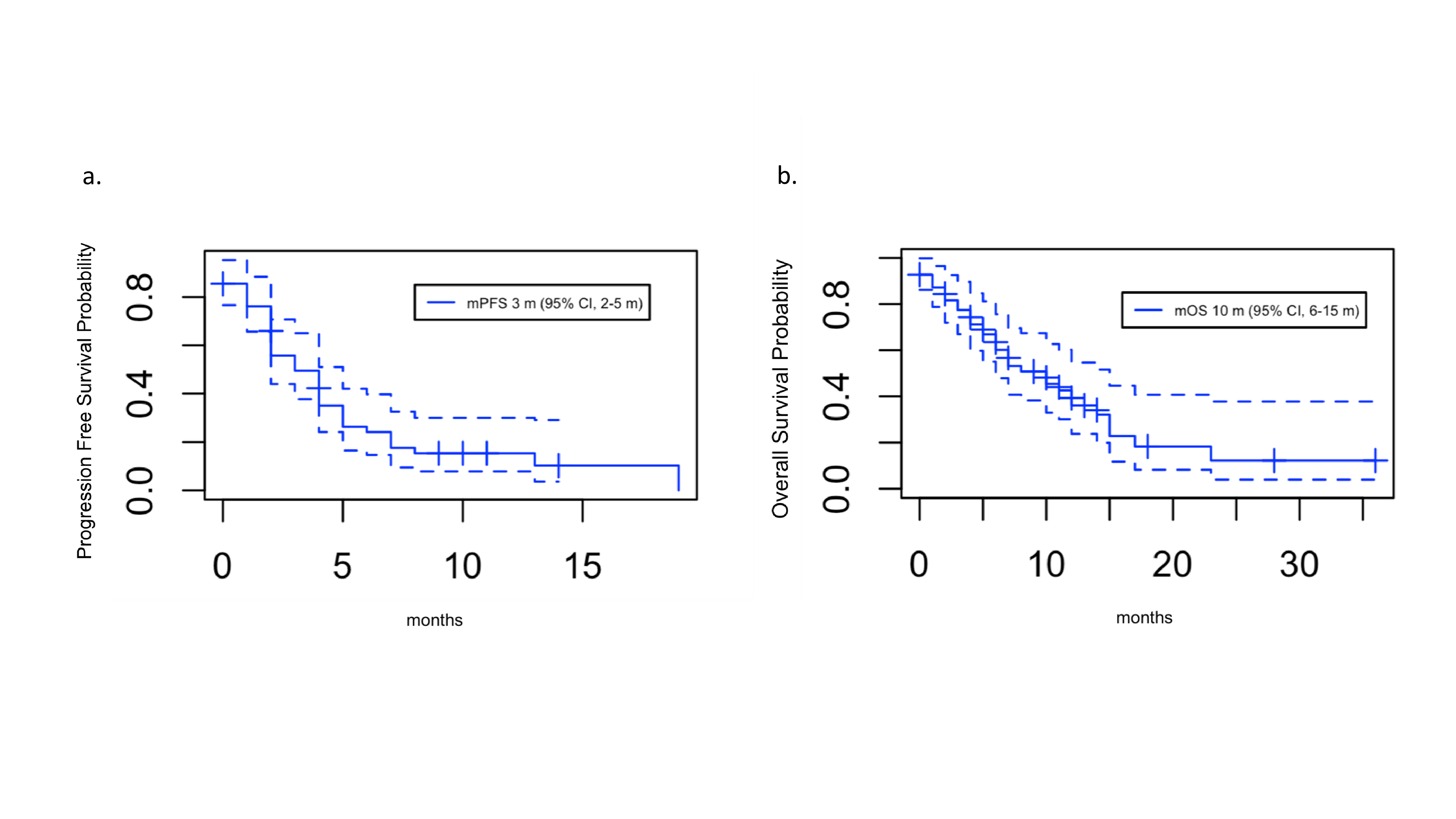

Supplement: Supplementary file 1 [file cancers-16-02589-s001.zip › Figure S2 egfr.tiff]
